# Does the optimal level of illumination improve both visual functions and visual comfort in schoolchildren with low vision?

**DOI:** 10.1371/journal.pone.0310592

**Published:** 2024-09-19

**Authors:** Pui Theng Yong, Zainora Mohammed, Norliza Mohamad Fadzil, Mohd Harimi Abd Rahman, Mohd Izzuddin Hairol, Sharanjeet Sharanjeet-Kaur, Sumithira Narayanasamy

**Affiliations:** 1 Centre for Rehabilitation and Special Needs Study, Faculty of Health Sciences, Universiti Kebangsaan Malaysia, Kuala Lumpur, Malaysia; 2 Centre for Community Health Studies, Faculty of Health Sciences, Universiti Kebangsaan Malaysia, Kuala Lumpur, Malaysia; Purdue University, UNITED STATES OF AMERICA

## Abstract

Lighting modification is commonly performed by optometrists and occupational therapists to enhance visibility and visual comfort among schoolchildren with low vision. The purpose of this study is to determine the optimal illumination level for visual function and visual comfort of schoolchildren with low vision and the relationship between them. A cross-sectional study was conducted to assess five levels of illumination ranging from 125 lux to 2000 lux to determine the optimal illumination for visual functions and visual comfort in schoolchildren with low vision from a special education school for blind in Malaysia. Purposive sampling was done to recruit forty-two schoolchildren with low vision for this study. Visual functions assessed were visual acuity, measured using the Early Treatment Diabetic Retinopathy Study LogMAR chart at distance and near, contrast sensitivity (CS) measured using the Pelli-Robson chart at distance and the Mars CS chart at near. Reading speed was determined using the Universiti Kebangsaan Malaysia Malay Language Related Word Reading Text test chart. Subjects were asked to rate their visual comfort using a validated questionnaire at the end of each measurement of visual functions and reading speed for the different illumination levels. Visual acuity and contrast sensitivity at distance and near, visual comfort and reading speed improved significantly with increase in illumination levels (p<0.05). However, the interaction between illumination level and level of low vision was not significant (p>0.05). Visual comfort was significantly associated with visual function (p<0.05), while direct association between visual comfort and illumination level was not significant (p>0.05). Optimal illumination for improvement of visual function, reading speed and visual comfort range from 276.67 lux to 701.59 lux. Majority of the schoolchildren with low vision had improved visual function, reading speed and visual comfort with increased illumination. Illumination of at least 600 lux is recommended for maximum visual functioning and visual comfort of schoolchildren with low vision.

## Introduction

Visual disturbance and visual fatigue caused by glaring and dim light are common complaints among people with low vision because of complication of various ocular diseases [1]. To maximise the remaining visual function, low vision aids such as magnifier, telescope and large print can be prescribed to enlarge the retinal image size and consequently improve reading performance [2]. In addition, modifying the illumination level is often recommended to further enhance the visual functions and eliminate photophobia [3–5]. However, the optimal illumination level for people with low vision has not been extensively investigated.

In Malaysia, the lighting requirements for specific tasks and buildings are guided by the Malaysia Standard (MS) and Standard and Industrial Research Institute of Malaysia (SIRIM). The lighting requirement recommended by the two agencies states that the classroom illuminance should be between 300 lux to 500 lux for ordinary reading and writing task, and 750 lux for art or technical drawings [6–8]. However, previous studies on patients with low vision found that higher illumination level is required to perform visual tasks effectively. A study conducted on elderly people with cataract found that that higher illumination level (1124 lux) was preferred over normal illumination [9]. Similar findings were reported for patient with aged-related macular degeneration [10]. It is argued that preference on higher illumination was related to concurrent increase in contrast sensitivity and visual resolution [11]. However, it is uncertain if visual function continues to improve with increasing illumination level [10, 12]. Although evidence showed that higher illumination could enhance vision, previous studies also found that lower illumination was preferable for visual comfort [13–15].

The optimal illumination for maximum visual function and visual comfort for children with low vision have not been reported. Relationship between illumination level, visual function, reading speed and visual comfort among schoolchildren with low vision has not been fully explored particularly among individuals with low vision. Ensuring appropriate lighting for schoolchildren with low vision is particularly important because they are often functioning at their visual threshold and improving illumination can improve their visual performance. Therefore, this study aims to determine the optimal illumination level for visual function and visual comfort of schoolchildren with low vision and the relationship between them.

## Materials and methods

This cross-sectional study was conducted from November 9, 2022 to April 29, 2023 at a special education school for visually impaired in Kuala Lumpur, Malaysia. Sample size of this study was calculated using formula for known population by Krejcie and Morgan (1970) [16], where minimum 37 subjects were required out of the total number of schoolchildren with low vision (n = 42) in the secondary special school to achieve statistical power of 0.80 at alpha level set at 0.50. All schoolchildren that had been identified as low vision through a comprehensive eye examination, were selected and invited to participate in this study through purposive sampling. According to the International Classification of Diseases 11th Revision (ICD-11), low vision is defined as best-corrected visual acuity (BCVA) of worse than 6/12 to 3/60 in the better eye. Low vision can be categorized as mild, moderate and severe, in which mild low vision has BCVA of worse than 6/12 to 6/18, worse than 6/18 to 6/60 for moderate low vision and worse than 6/60 to 3/60 for severe low vision in the better eye [17]. Besides, the sampling criteria includes (i) age between 13 to 18 years, (ii) vision worse than 6/12 to 3/60 and (iii) absence of cognitive disabilities as indicated in the medical record and database that are available at the school and the Ministry of Education Malaysia information system. This study has been approved by the Department of Special Education and Ministry of Education Malaysia, and the Universiti Kebangsaan Malaysia Research Ethics Committee [UKM PPI/111/8/JEP-2022-560]. Written informed consent from the participants and their parent/ guardian were obtained through the school administrative staff.

**Fig 1:**
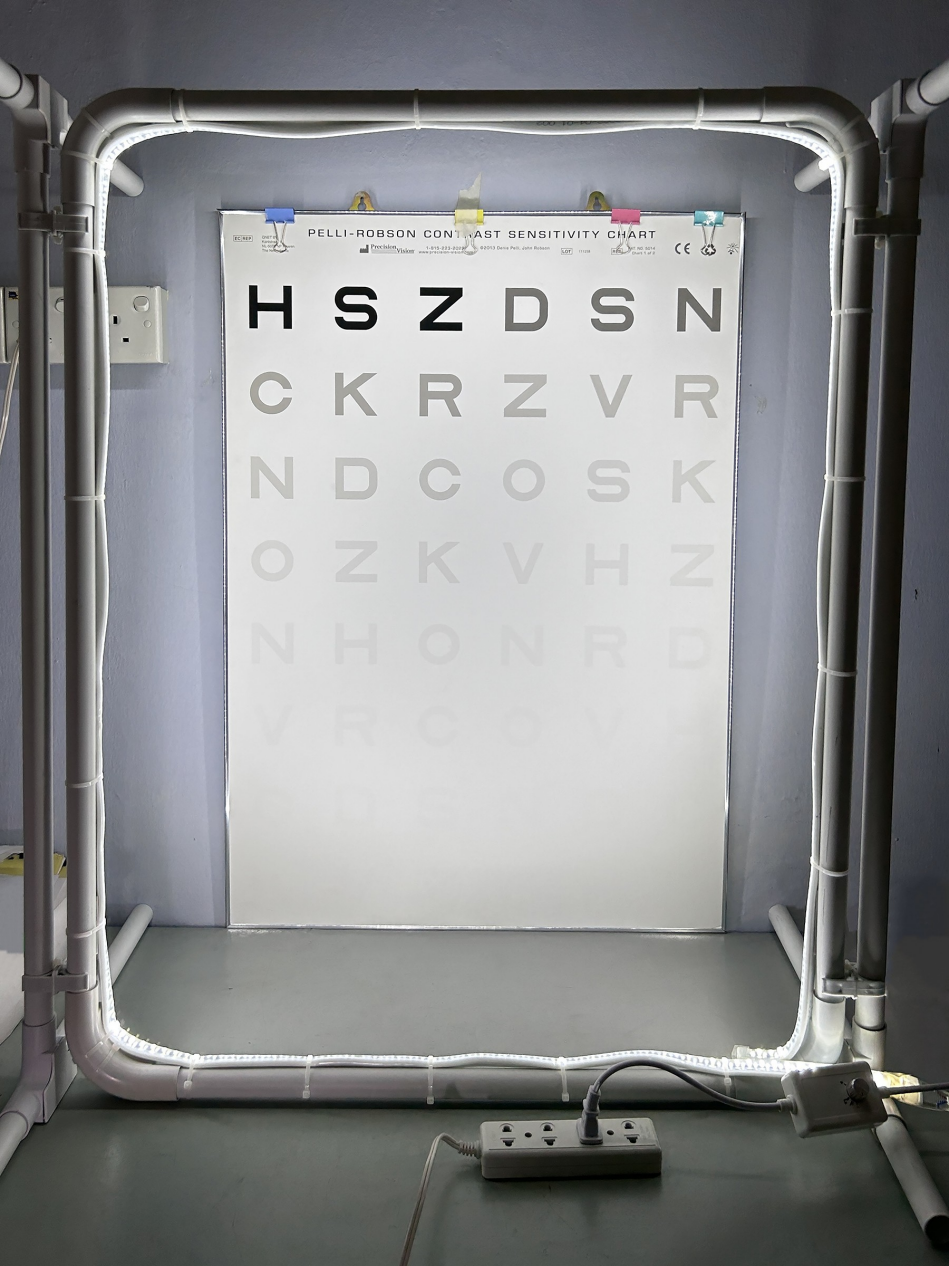
LED light frame with Pelli-Robson chart.

The study was conducted in a room that was set-up at the school. Illumination level was measured using lux meter model ILM1335 (ISO-TECH, Taiwan). The room illumination level when all lights were switch off and the windows were covered with blinds was approximately 30 lux. Five illumination levels of 125 lux, 250 lux, 500 lux, 1000 lux and 2000 lux were used in this study. These illumination levels were determined by placing the lux meter on the surface of charts with the light sensor facing upwards towards the light source. Five measurements were made which were at each corner as well as the centre of the charts. The acceptable measurement at each illumination levels were set within ±10 lux. For distance visual tasks, light frame installed with light-emitting diode (LED) strip and electric dimmer was used to provide the varying levels of illumination (Fig 1). For near visual tasks, the Xiaomi smart LED bulb with dimmer was installed in a light bulb stand and placed 25 cm from the tasks. Both LED light strip and LED light bulb colour temperature were 6000 Kelvin (K) to mimic natural daylight condition [18]. Distance visual acuity was measured using the Early Treatment Diabetic Retinopathy Study (ETDRS) LogMAR chart (Precision Vision Inc. Illinois, USA) at 2 m and contrast sensitivity was measured using Pelli-Robson chart (Precision Vision Inc. Illinois, USA) at 1 m. Near visual acuity was measured using the ETDRS near chart at 40 cm. Reading speed was measured at 40 cm using the Universiti Kebangsaan Malaysia Malay Language Related Word Reading Text Test chart level 2, which is suitable for children aged 10 and above [19]. The measurements of visual function (distance visual acuity, distance contrast sensitivity, near visual acuity and near contrast sensitivity), reading speed and visual comfort was explained. The distance and near visual acuity of the subjects are defined as their visual threshold as that is their smallest recognizable print size. The Universiti Kebangsaan Malaysia Malay Language Related Word Reading Text Test chart consists of seven to eight lines of words and each line of words not exceeding 60 characters including blank spaces. There were five test charts with different words combination. The subjects were told to read aloud the reading test chart at their habitual speed. The examiner started the timer and concurrently checked the accuracy of their reading. Error in reading were defined as incorrectly read or omitted words. Reading speed is determined by dividing the number of words read correctly to the time (in second, s) taken to complete the paragraph, and converting this to words per minute (wpm) using the following formula [20]:

$$Reading\;speed\;(word\;per\;minute,wpm)=\frac{Number\;of\;words\;read\;correctly}{Time\;used\;to\;finish\;reading\;(s)}\times 60$$

Near contrast sensitivity was measured using the Mars contrast sensitivity test card at 50 cm.

Refractive error of the subjects was determined using auto-refractometer followed by subjective refraction. The study was conducted using their best refractive correction. Prior to the study, subjects were seated under a specified lighting (125 lux, followed by 250 lux, 500 lux, 1000 lux and 2000 lux for each step) for five minutes while reading an article of standard letter size. This procedure was to ensure that equilibrium of room’s luminance and subject’s lighting adaptation was achieved. The study always started with measurement of distance visual functions followed by near visual functions and reading speed at each illumination levels from 125 to 2000 lux. Different set of charts were used to minimize practice effect, and subjects were asked to rest for ten to fifteen minutes between measurements to avoid fatigue. Subjects were required to rate their visual comfort using a validated Malay-version Visual Comfort Questionnaire after each visual function or reading test at each illumination levels. The questionnaire was adapted from a study by Lee et al. (2014) [14], which was translated and validated to Malay language in another phase of this study. The Malay version of Visual Comfort Questionnaire has acceptable Fleiss’ Kappa inter-rater reliability of relevance (κ = 0.54) and comprehensibility (κ = 0.44), as well as good internal consistency (Cronbach’s alpha = 0.96). It consists of fourteen items and seven levels of Likert scale from strongly disagree to strongly agree. Visual Comfort Questionnaire was self-administered for each participant to rate each item as strongly disagree (1), disagree (2), uncertain or neutral (3), agree (4), and strongly agree (5) according to their perception on each statement.

### Statistical analysis

IBM SPSS Statistics version 26 (IBM Corp., Armonk, N.Y., USA) was used to conduct the analyses including Mixed Model ANOVA, Friedman test, Kruskal-Wallis test, follow-up pairwise comparison tests, Quade’s non-parametric ANCOVA test and Kendall’s tau-b test. Igor Pro® (WaveMetrics®, Portland, O.R., USA) was used to graph the log changes of all parameters across the five illumination levels with best-fit line and determine the critical value for the peak of the best-fitting line.

## Results

A total of 42 schoolchildren with low vision were examined from October 2022 to February 2023. There were 24 (57.1%) male and 18 (42.9%) female subjects, aged from 13 to 18 years (M = 14.90, SD = 1.27). Among the subjects, four (9.5%) of them had mild low vision (6/12 to 6/18), 30 (71.4%) had moderate low vision (<6/18 to 6/60), and eight (19.0%) had severe low vision (<6/60 to 6/120). The demographic data of the subjects are shown in Table 1.

**Table 1:** Demographic data of subjects.

Demographic parameters	Frequency	Percentage
**Gender**		
Male	24	57.1%
Female	18	42.9%
**Age**		
13–14 years	15	35.7%
15–16 years	25	59.5%
17–18 years	2	4.8%
**Level of low vision**		
Mild (6/12–6/18)	4	9.5%
Moderate (<6/18–6/60)	30	71.4%
Severe (<6/60–6/120)	8	19.0%
**Cause of low vision**		
Optic nerve disease	9	21.4%
Retinal/ macular disease	8	19.0%
Amblyopia/ tropia	7	16.7%
Nystagmus	6	14.3%
Cataract	4	9.5%
Iris lesion	2	4.8%
Albinism	1	2.4%
High myope	1	2.4%
Unknown	4	9.5%
**Better eye**		
Right	19	45.2%
Left	23	54.8%
**Corrective spectacle**		
Yes	23	45.2%
No	19	54.8%

Data from one eye which is the best eye or right eye (if both eyes have similar measurements) was used in the analysis.

### Effect of illumination level on visual functions and visual comfort in schoolchildren with mild, moderate and severe low vision

The mean and standard deviation of measurements made at 125 lux, 250 lux, 500 lux, 1000 lux and 2000 lux were determined for each visual functions and reading speed in schoolchildren with mild, moderate and severe low vision (Table 2).

**Table 2:** Mean ± standard deviation of visual function, reading speed and visual comfort of subjects with mild, moderate and severe low vision under different illumination level.

Parameters	Level of low vision	125 lux	250 lux	500 lux	1000 lux	2000 lux
**Distance visual acuity (logMAR)**	Mild	0.41 ±0.13	0.36 ±0.06	0.31 ±0.14	0.36 ±0.06	0.32 ±0.12
	Moderate	0.74 ±0.21	0.71 ±0.19	0.70 ±0.19	0.69 ±0.19	0.68 ±0.20
	Severe	0.90 ±0.25	0.87 ±0.27	0.84 ±0.31	0.83 ±0.33	0.81 ±0.33
	Average	0.74 ±0.24	0.71 ±0.24	0.69 ±0.25	0.68 ±0.24	0.67 ±0.25
**Near visual acuity (logMAR)**	Mild	0.47 ±0.11	0.41 ±0.08	0.34 ±0.10	0.35 ±0.10	0.37 ±0.11
	Moderate	0.76 ±0.19	0.72 ±0.21	0.70 ±0.21	0.69 ±0.22	0.70 ±0.22
	Severe	0.87 ±0.38	0.83 ±0.38	0.78 ±0.32	0.79 ±0.31	0.77 ±0.31
	Average	0.76 ±0.25	0.71 ±0.26	0.68 ±0.25	0.68 ±0.25	0.68 ±0.25
**Distance contrast sensitivity (logCS)**	Mild	1.33 ±0.43	1.50 ±0.53	1.53 ±0.54	1.49 ±0.55	1.51 ±0.54
	Moderate	1.01 ±0.40	1.15 ±0.40	1.21 ±0.37	1.24 ±0.37	1.23 ±0.38
	Severe	0.60 ±0.55	0.79 ±0.50	0.80 ±0.49	0.81 ±0.48	0.77 ±0.40
	Average	0.96 ±0.47	1.12 ±0.46	1.16 ±0.45	1.18 ±0.44	1.17 ±0.44
**Near contrast sensitivity (logCS)**	Mild	1.46 ±0.42	1.50 ±0.43	1.55 ±0.40	1.52 ±0.38	1.55 ±0.40
	Moderate	1.15 ±0.35	1.24 ±0.36	1.30 ±0.36	1.33 ±0.36	1.31 ±0.35
	Severe	0.87 ±0.55	0.91 ±0.56	1.00 ±0.53	1.04 ±0.48	1.02 ±0.46
	Average	1.12 ±0.42	1.20 ±0.43	1.26 ±0.42	1.29 ±0.40	1.28 ±0.39
**Reading speed (wpm)**	Mild	87.50 ±25.63	104.75 ±32.73	105.00 ±29.16	101.00 ±28.76	106.50 ±39.75
	Moderate	48.30 ±23.91	56.43 ±29.50	59.43 ±30.15	61.73 ±33.58	62.80 ±34.45
	Severe	38.38 ±21.70	47.63 ±26.92	50.50 ±24.63	49.75 ±22.95	52.00 ±28.57
	Average	50.14 ±26.43	59.36 ±32.43	62.07 ±31.94	63.19 ±33.53	64.90 ±36.04
**Visual comfort (linear score)**	Mild	53.50 ±16.90	55.00 ±12.06	53.75 ±14.50	54.25 ±21.72	46.00 ±22.00
	Moderate	43.70 ±11.46	50.03 ±12.20	54.00 ±10.51	57.63 ±10.36	55.73 ±12.74
	Severe	48.00 ±8.11	55.00 ±9.77	54.38 ±7.67	57.25 ±10.71	57.13 ±12.58
	Average	45.45 ±11.61	51.45 ±11.72	54.05 ±10.18	57.24 ±11.44	55.07 ±13.66

From Table 2, it can be seen that data of the parameters measured varies considerably, probably due to the variation of diseases causing visual loss. Therefore, to overcome individual variation, relative change rather than absolute change was used to determine subject’s performance at different illumination levels. The use of relative change, where the baseline data was set at the lowest illumination level (125 lux), allow comparison to be made across different scales of parameters. For consistency of comparison (statistically and visually using graph), log conversion of reading speed and visual comfort were made as visual acuity and contrast sensitivity were measured in log unit.

### Effect of Illumination levels on distance and near visual acuity

Table 3 shows the changes (in log difference) of distance and near visual acuity at 250 lux, 500 lux, 1000 lux and 2000 lux compared to 125 lux (baseline). Mixed model ANOVA (5 x 3) was used to determine the effect of illumination level on distance and near visual acuity. Results of main effect showed a significant reduction of logMAR distance visual acuity as illumination level increased, *F* (2.35, 156) = 9.94, *p* <0.05, partial *η*2 = 0.20. Level of low vision has no significant effect on distance visual acuity, *F* (2, 39) = 0.29, *p* = 0.75. The interaction between illumination level and level of low vision was not significant, *F* (4.70, 156) = 0.72, *p* = 0.60. For near visual acuity, mixed model ANOVA (5 x 3) indicated that visual acuity significantly reduced as illumination level increased, *F* (2.51, 156) = 15.49, *p* <0.05, partial *η*2 = 0.28. Collapsed across illumination level, level of low vision has no significant effect on near visual acuity, *F* (2, 39) = 0.56, *p* = 0.58. Interaction between illumination level and level of low vision was not significant, *F* (5.02, 156) = 0.82, *p* = 0.54.

**Table 3:** Mean and standard deviation of changes (log difference) in visual function, reading speed and visual comfort of subjects with mild, moderate and severe low vision at different illumination level compared to baseline.

Parameters	Level of low vision	250 lux	500 lux	1000 lux	2000 lux
Distance visual acuity (logMAR)	Mild	-0.05 ±0.11	-0.11 ±0.18	-0.05 ±0.13	-0.10 ±0.16
	Moderate	-0.03 ±0.05	-0.05 ±0.06	-0.06 ±0.07	-0.06 ±0.07
	Severe	-0.03 ±0.06	-0.06 ±0.09	-0.07 ±0.12	-0.09 ±0.12
	Average	-0.03 ±0.06	-0.05 ±0.08	-0.06 ±0.09	-0.07 ±0.09
Near visual acuity (logMAR)	Mild	-0.06 ±0.06	-0.13 ±0.48	-0.12 ±0.23	-0.10 ±0.02
	Moderate	-0.05 ±0.07	-0.07 ±0.08	-0.07 ±0.08	-0.07 ±0.09
	Severe	-0.05 ±0.05	-0.09 ±0.10	-0.08 ±0.10	-0.08 ±0.10
	Average	-0.05 ±0.06	-0.08 ±0.08	-0.08 ±0.08	-0.08 ±0.10
Distance contrast sensitivity (logCS)	Mild	0.18 ±0.29	0.20 ±0.27	0.16 ±0.31	0.19 ±0.28
	Moderate	0.14 ±0.13	0.20 ±0.17	0.23 ±0.19	0.22 ±0.24
	Severe	0.19 ±0.16	0.20 ±0.28	0.21 ±0.34	0.17 ±0.34
	Average	0.15 ±0.15	0.20 ±0.20	0.22 ±0.23	0.21 ±0.26
Near contrast sensitivity (logCS)	Mild	0.04 ±0.03	0.09 ±0.02	0.06 ±0.05	0.09 ±0.04
	Moderate	0.08 ±0.11	0.15 ±0.13	0.18 ±0.13	0.16 ±0.16
	Severe	0.04 ±0.06	0.13 ±0.09	0.17 ±0.20	0.15 ±0.29
	Average	0.07 ±0.10	0.14 ±0.12	0.17 ±0.14	0.15 ±0.18
Reading speed (log decimal)	Mild	0.20 ±0.12	0.21 ±0.15	0.17 ±0.17	0.21 ±0.21
	Moderate	0.17 ±0.14	0.24 ±0.19	0.28 ±0.23	0.30 ±0.27
	Severe	0.22 ±0.19	0.44 ±0.50	0.48 ±0.70	0.51 ±0.70
	Average	0.18 ±0.15	0.27 ±0.28	0.30 ±0.36	0.33 ±0.38
Visual comfort (log decimal)	Mild	0.07 ±0.21	0.09 ±0.39	0.15 ±0.63	-0.04 ±0.49
	Moderate	0.17 ±0.21	0.32 ±0.45	0.45 ±0.59	0.41 ±0.66
	Severe	0.15 ±0.11	0.15 ±0.13	0.21 ±0.20	0.21 ±0.28
	Average	0.15 ±0.19	0.26 ±0.41	0.37 ±0.54	0.33 ±0.60

Pairwise comparison was conducted to determine the significant difference of distance and near visual acuity at different illumination level. After adjusted the alpha level with Bonferroni correction, the distance visual acuity at 500 lux (*p* <0.01) and 2000 lux (*p* <0.01) was significantly different from 125 lux (baseline), while near visual acuity at 250 lux (*p* <0.01), 500 lux (*p* <0.01) and 1000 lux (*p* <0.01) were significantly different from 125 lux.

### Effect of illumination level on distance and near contrast sensitivity

Changes (in log difference) of distance and near contrast sensitivity at 250 lux, 500 lux, 1000 lux and 2000 lux compared to 125 lux (baseline) are shown in Table 3. Mixed model ANOVA (5 x 3) was conducted to investigate the effect of illumination level on distance and near contrast sensitivity. The results showed that, distance contrast sensitivity increased significantly across the five illumination levels, *F* (2.08, 156) = 10.41, *p* <0.05, partial *η*2 = 0.21. The between-subject factor, level of low vision has no significant effect on the distance contrast sensitivity, *F* (2, 39) = 0.01, *p* = 0.99. The interaction between illumination level and level of low vision is not significant, *F* (4.15, 156) = 0.41, *p* = 0.81. The main effect showed that, near contrast sensitivity increased significantly as illumination level increased, *F* (2.15, 156) = 10.28, *p* <0.05, partial *η*2 = 0.21. Level of low vision has no significant effect on near contrast sensitivity, *F* (2, 39) = 0.70, *p* = 0.50. The interaction between illumination level and level of low vision is not significant, *F* (4.29, 156) = 0.58, *p* = 0.69.

Pairwise comparison to determine the significance of difference between contrast sensitivity under different illumination level at distance and near was conducted. After adjusted the alpha level using Bonferroni correction, the distance contrast sensitivity at illumination level of 250 lux (*p* <0.01), 500 lux (*p* <0.01) and 1000 lux (*p* <0.01) were significantly different compared to 125 lux (baseline), while near contract sensitivity at illumination level of 500 lux (*p* <0.01) and 1000 lux (*p* <0.01) were significantly different compared to 125 lux (baseline).

### Effect of Illumination level on reading speed

Table 2 shows the reading speed (wpm) at 125 lux, 250 lux, 500 lux, 1000 lux and 2000. Normality test reported a not normal distribution of data for reading speed (wpm). Therefore, Friedman test was conducted to determine the difference of reading speed (wpm) across the five illumination levels, including 125 lux, 250 lux, 500 lux, 1000 lux and 2000 lux. Results showed that, reading speed (wpm) significantly increased across the illumination levels, $X_{F}^{2}$ = 73.18 (corrected for ties), *df* = 4, N–Ties = 42, *p* <0.05. Follow-up pairwise comparisons was conducted with Wilcoxon Signed Rank test, while results showed significant different of 250 lux (*p* <0.01), 500 lux (*p* <0.01), 1000 lux (*p* <0.01) and 2000 lux (*p* <0.01) from 125 lux.

Kruskal-Wallis test was used to investigate the between subject effect. Results indicated that, there was a significant difference of reading speed (wpm) among the schoolchildren with different levels of low vision, [*H* (corrected for ties) = 33.71, *df* = 2, N = 210, *p* <0.05]. Follow-up pairwise comparison was conducted using Mann-Whitney *U* test. After adjusted the alpha level with Bonferroni correction, results indicated that the reading speed (wpm) of schoolchildren with mild low vision (*Mean Rank* = 141.65, *n* = 20) were significantly higher compared to schoolchildren with moderate low vision (*Mean Rank* = 78.01, *n* = 150), *U* = 377.00, *z* = –5.43 (correct for ties), p <0.017, two-tailed. A significantly higher reading speed (wpm) was seen among schoolchildren with mild low vision compared to schoolchildren with severe low vision (*Mean Rank* = 22.01, *n* = 40), *U* = 60.50, *z* = –5.33 (correct for ties), *p* <0.017, two-tailed. However, there was no significant difference in reading speed (wpm) between schoolchildren with moderate low vision and severe low vision, *U* = 2589.00, *z* = –1.33 (correct for ties), *p* = 0.183, two-tailed.

Table 3 shows the relative changes (in log difference) of reading speed at 250 lux, 500 lux, 1000 lux and 2000 lux compared to 125 lux (baseline). Friedman test was conducted to determine the effect of illumination level on the reading speed (log decimal) because the data was not normally distributed. Results showed that, reading speed (log decimal) significantly increased across the illumination levels, $X_{F}^{2}$ = 73.18 (corrected for ties), *df* = 4, N–Ties = 42, *p* <0.05. Follow-up pairwise comparisons was conducted with Wilcoxon Signed Rank test. Results showed significant different of 250 lux (*p* <0.01), 500 lux (*p* <0.01), 1000 lux (*p* <0.01) and 2000 lux (*p* <0.01) from the baseline.

On the other hand, results of Kruskal-Wallis test showed no significant difference on the relative changes of reading speed (log decimal) among schoolchildren with mild low vision, moderate low vision and severe low vision [*H* (corrected for ties) = 0.67, *df* = 2, N = 210, *p* = 0.72].

### Effect of illumination level on visual comfort

Changes (in log difference) of visual comfort at 250 lux, 500 lux, 1000 lux and 2000 lux compared to 125 lux (baseline) are shown in Table 3. Friedman test was conducted to determine the effect of illumination level among the subjects with low vision. The results showed that visual comfort significantly increased as illumination level increased, $X_{F}^{2}$ = 64.52 (corrected for ties), *df* = 4, N–Ties = 42, *p* <0.05. Follow-up pairwise comparison using Wilcoxon Signed Rank test showed that, visual comfort at 250 lux (*p* <0.01), 500 lux (*p* <0.01), 1000 lux (*p* <0.01) and 2000 lux (*p* <0.01) was significantly different from the baseline.

Kruskal-Wallis test was used to determine the effect of level of low vision on visual comfort. Results showed that the different level of low vision has no significant effect on visual comfort, *H* (corrected for ties) = 2.64, *df* = 2, N = 210, *p* = 0.27.

Table 4 shows the statistical findings of distance and near visual acuity, distance and near contrast sensitivity, reading speed, and visual comfort among the schoolchildren with low vision.

**Table 4:** Statistical findings of visual functions, reading speed and visual comfort.

Parameter	Statistical test		Statistical finding	Significance
Distance visual acuity	Mixed model ANOVA	Within group	*F* (2.35, 156) = 9.94	*p* < 0.05
		Between group	*F* (2, 39) = 0.29	*p* = 0.75
Near visual acuity	Mixed model ANOVA	Within group	F (2.51, 156) = 15.49	*p* < 0.05
		Between group	*F* (2, 39) = 0.56	*p* = 0.58
Distance contrast sensitivity	Mixed model ANOVA	Within group	*F* (2.08, 156) = 10.41	*p* < 0.05
		Between group	*F* (2, 39) = 0.01	*p* = 0.99
Near contrast sensitivity	Mixed model ANOVA	Within group	*F* (4.15, 156) = 0.41	*p* < 0.05
		Between group	*F* (2, 39) = 0.70	*p* = 0.50
Reading speed (wpm)	Friedman test		$X_{F}^{2}$ = 73.18 (corrected for ties)	*p* < 0.05
	Kruskal-Wallis test		*H* (corrected for ties) = 33.71	*p* <0.05
Reading speed (log decimal)	Friedman test		$X_{F}^{2}$ = 73.18 (corrected for ties)	*p* < 0.05
	Kruskal-Wallis test		*H* (corrected for ties) = 0.67	*p* = 0.72
Visual comfort	Friedman test		$X_{F}^{2}$ = 64.52 (corrected for ties)	*p* < 0.05
	Kruskal-Wallis test		*H* (corrected for ties) = 2.64	*p* = 0.27

### Association between visual function, reading speed and visual comfort

Due to the not-normally distributed data, Quade non-parametric ANCOVA was conducted to identify the relationship between visual function and visual comfort. Results showed that, the direct relationship between visual comfort and illumination level was not significant after diminished effect of visual functions, *F* (4, 205) = 1.04, *p* = 0.39.

The association between visual comfort, reading speed and each visual function was determined using Kendall’s tau-b correlation. All visual functions showed significant association with visual comfort (distance visual acuity, *r* = -0.38, *p* <0.05; near visual acuity was significant, *r* = -0.32, *p* <0.05; distance contrast sensitivity, *r* = 0.42, *p* <0.05; near contrast sensitivity, *r* = 0.42, *p* <0.05). Kendall’s tau-b correlation test also showed significant association between visual comfort and reading speed, *r* = 0.34, *p* <0.05.

Kendall’s tau-b correlation also showed significant association between reading speed and near visual acuity, *r* = -0.37, *p* <0.05 and significant association between reading speed and near contrast sensitivity, *r* = 0.34, *p* <0.05.

### Optimal illumination level for visual function, reading speed and visual comfort

**Fig 2:**
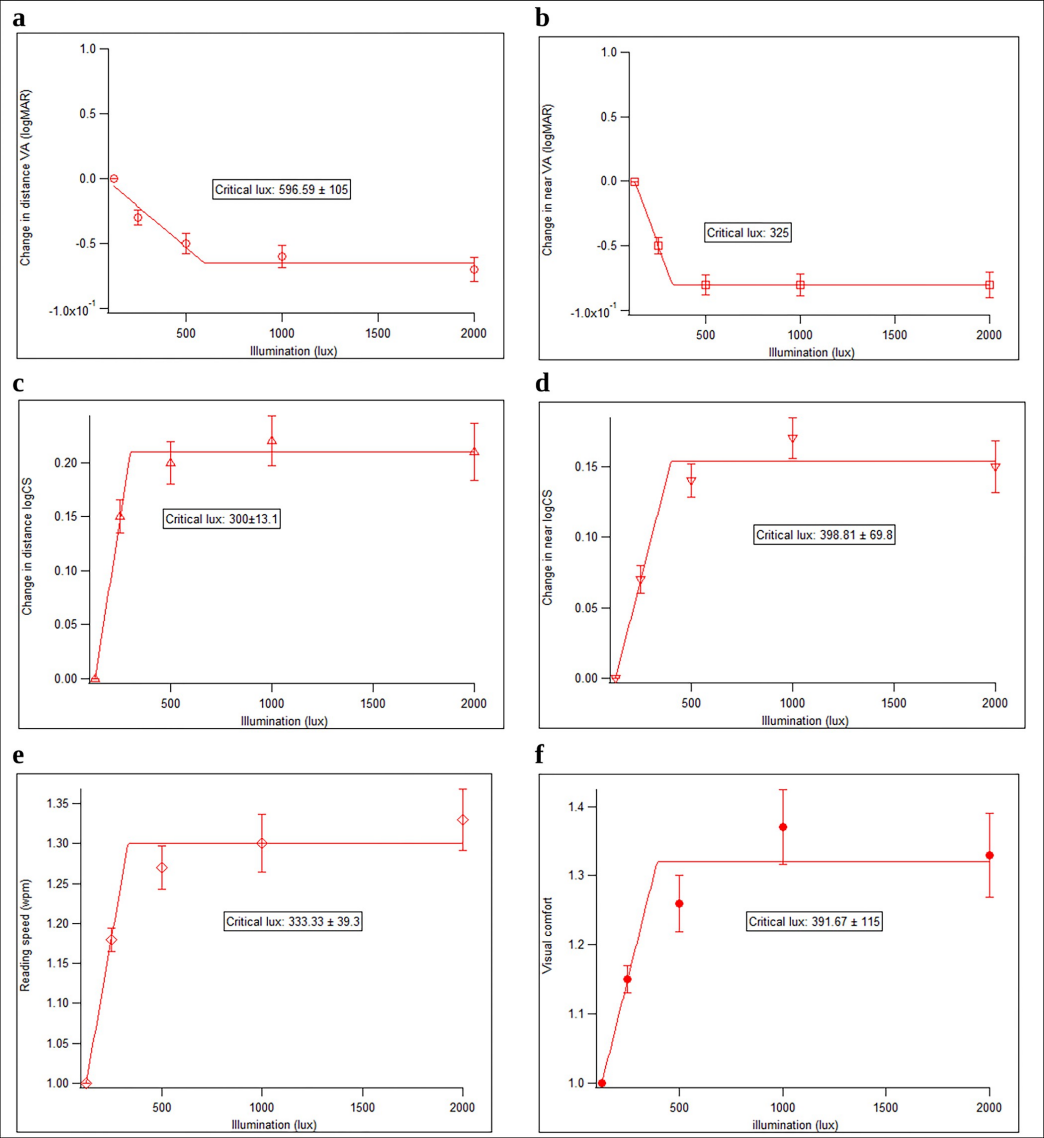
Change of visual function, reading speed and visual comfort across five illumination levels. (a) distance visual acuity. (b) near visual acuity. (c) distance contrast sensitivity. (d) near contrast sensitivity. (e) reading speed. (f) visual comfort.

Optimal illumination level is referred to the level at which highest functioning is achieved either in terms of visual acuity, contrast sensitivity, reading speed or visual comfort. The graph with best-fitting line of distance visual acuity, near visual acuity, distance contrast sensitivity, near contrast sensitivity, reading speed and visual comfort across the five illumination level were plotted. Then, the critical value of illumination level was determined based on the best-fitting line (y_i_ = b_0_ + b_1_x_i_) for each parameter. Critical illumination level is the minimum illumination level that leads to better performance for tasks. The critical illumination level was 596.59 ± 105.00 lux for distance visual acuity, 325.00 lux for near visual acuity, 300.00 ± 13.10 lux for distance contrast sensitivity, 398.81 ± 69.80 lux for near contrast sensitivity, 333.33 ± 39.30 lux for reading speed and 391.67 ± 115.00 lux for visual comfort. The average critical illumination level for all parameters ranged from 276.67 lux to 701.59 lux. In general, the optimal illumination is 600 lux for all tasks, based on the judgement on graphical patterns for changes of visual function, reading speed and visual comfort across the five illumination level. Fig 2 shows the graph plotting the changes of distance visual acuity, near visual acuity, distance contrast sensitivity, near contrast sensitivity, reading speed and visual comfort across the five illumination levels.

Majority of the subjects showed improvement with increase illumination levels in all the parameters measured. According to the frequency of schoolchildren with low vision achieving their optimal state of visual function, reading speed and visual comfort for each illumination level, majority (>50%) of subjects achieved maximum distance visual acuity and contrast sensitivity at 1000 lux and above, maximum near visual acuity and contrast sensitivity at 500 lux and above, maximum reading speed at 2000 lux and visual comfort at 1000 lux. It should also be noted that frequency of subjects that can achieved maximum distance contrast sensitivity and visual comfort reduced at 2000 lux. However, some of the schoolchildren with low vision had optimal visual function, reading speed and visual comfort at lower illumination level.

## Discussion

This study seeks to determine the optimal illumination level for visual acuity, contrast sensitivity, reading performance and visual comfort of schoolchildren with low vision and the relationship between them. The measure of visual function such as visual acuity and contrast sensitivity describe the function of the eye and visual system. To understand how the schoolchildren with low vision perform in vision related activities, a measure of functional vision is necessary. In this study, reading speed is used because reading is one of the main learning activities in school, and it can be affected by reduced visual acuity and contrast sensitivity. It was found that schoolchildren with low vision showed significant improvement in visual acuity, contrast sensitivity, reading speed and visual comfort with increased illumination level, regardless of their level of low vision or cause of vision loss. The optimal illumination level for each parameter varied, where optimal illumination level of 596.59 ± 105.00 lux is required for distance visual acuity, while approximately 300 lux is required for near visual acuity, distance and near contrast sensitivity, reading speed and visual comfort. It can be generalised that the optimal illumination level for schoolchildren with low vision population was 600 lux for better visual function, reading speed and visual comfort. During the statistical analysis, significant improvements of visual function, reading speed and visual comfort across the illumination level were reported, but statistically significant values does not reflect the importance of lighting modifications in real life environments. The illumination levels in this study were task lighting and did not consider ambient lighting. According to the frequencies of schoolchildren with low vision with maximized visual function, reading speed and visual comfort under each illumination level, illumination level of 1000 to 2000 lux were more preferrable for majority of schoolchildren with low vision. Thus, this study underscores the benefits of providing appropriate level of illumination to improve the visibility of visual tasks. Alternatively, use of electronic devices that allow higher levels of contrast and magnification can be utilized, and this approach may be more attractive to the younger generation who are familiar with the use of gadgets. In this study, the type of optical devices prescribed to the schoolchildren was not determined. However, a previous study done at the same school reported that only five percent of the schoolchildren used electronic devices whilst the remaining were prescribed with simple optical devices such as handheld and stand magnifier [21].

The optimal illumination level determined in this study was higher for children with low vision than recommended classroom illumination by Malaysia Standard MS 1525:2014, in which illumination level between 300 lux to 500 lux are recommended for reading and writing tasks in ordinary classroom, while 750 lux is recommended for specific tasks that require detailed and precise vision, such as art class and technical drawing class [6]. Classroom illumination level of 300 lux to 500 lux are also recommended by other countries for children with normal vision [22]. The Illuminating Engineering Society of North America (IESNA) provided a standard of illuminance for Unites States of America in the IES handbook, where a range within 300 lux to 500 lux was suggested illuminance in the typical classroom. The recommended illuminance for the classrooms in the United Kingdom was also 300 lux to 500 lux according to the Code of Interior Lighting. According to the Singapore Standard SS 531: 2006 established by the Technical Committee on Lamps and Related Equipment supervised by the Electronic Standards Committee, illuminance from 300 lux to 500 lux was suggested for the classroom. Other than that, the Illuminating Engineering Society of Chinas suggested a 300 lux of illumination level for the classrooms, while illumination between 250 lux to 300 lux was recommended by Indonesia National Standard SNI 03-6197-2000 [21]. These standards were schemed based on the normal population, but our study findings showed a higher illumination level to optimize the visual function, reading speed and visual comfort of schoolchildren with low vision in classroom. Table 5 shows the standard illumination level recommended for classroom at different countries, developed based on the normal population.

**Table 5:** Standard illumination level recommended for classroom at different countries.

Department/ Standard	Country	Recommended illumination level (lux)
Department of Standards Malaysia (MS 1525:2014)	Malaysia	300 to 500
Illuminating Engineering Society of North America (ANSI/IES RP-3-20)	United States	300 to 500
Chartered Institution of Building Services Engineers (SLL Code for Lighting)	United Kingdom	300 to 500
Electronic Standards Committee (SS 531: 2006)	Singapore	300 to 500
China Illuminating Engineering Society (GB 7793–2010)	China	300
Indonesia National Standard (SNI 03-6197-2000)	Indonesia	250 to 300

To the best of our knowledge, this is the first study to determine the optimal illumination level for assessing reading speed among schoolchildren with low vision. Previous studies particularly focused on the illumination level that optimize the visual function and preferred illumination level that optimize visual clarity and visual comfort among individuals with low vision [9, 23]. However, previous findings did not define a range of optimal illumination levels that benefit all schoolchildren with low vision with different lighting needs in the same classroom.

The findings of illumination level that optimized visual acuity and visual comfort were supported by previous studies [9, 23]. That is, preferred illumination level for majority of people with low vision are higher than the recommended illumination level for normal population. Henry et al. (2020) conducted a study to compare the near visual acuity under 750 lux and 1000 lux among visually impaired adults. Their results showed that near visual acuity improved with 1000 lux, and the subjects also reported that 1000 lux is preferable for reading task [23]. Evan et al. (2010) conducted a study to determine the illumination level that produced clear vision and comfort for reading task among adults with cataract and aged-related macular degeneration. Their results showed that, the mean preferred illumination level of subjects with cataract were 1134 ±900 lux for clear vision and 936 ±802 lux for comfort, while subjects with aged-related macular degeneration preferred 990 ±212 lux for clear vision and 715 ±275 lux for comfort [9]. However, visual comfort in our study showed contrasting findings compared to an earlier study on effect of illumination level on the visual comfort and among normal sighted university students. In Avcı and Memikoğlu (2021) study, illumination level of 500 lux was preferrable for visual comfort, followed by 200 lux and 800 lux [13]. This finding indicates that, higher illumination level was less likely to give optimal visual comfort compared to lower illumination levels. However, the subjects in our study were low vision, which may be one of the factors where results showed a higher illumination level was preferred for optimal visual function, reading speed and visual comfort.

Illumination level for optimal reading speed is higher than other visual functions among the majority of schoolchildren with low vision. This could be due to most of the subjects had optimal near visual acuity (n = 27, 64%) and optimal near contrast sensitivity (n = 26, 62%) at 2000 lux. This finding was contraindicated with previous studies, where illumination level was not consistently related to reading speed when the reading task does not involve small letter size [23–25]. This is probably due to the reading chart provided to subjects in this study had similar text size as their near visual acuity, which is their visual acuity threshold. However, this study finding shows similar finding with an earlier study conducted to determine the reading performance of older adults with age-related macular degeneration under different illumination levels [12]. Bowers, Meek, Stewart (2001) [12] reported a significant increase in near visual acuity from 50 lux to 2000 lux among the subjects. However, there were significant difference between measurements done by standard near vision chart and sentence near vision chart. In which, further improvement in near visual acuity from 2000 lux to 5000 lux were reported when measurements were done using near vision chart with sentence/ string [12]. However, reading speed improved only up to 1000 lux [12]. This is due to the fading of vision among the older adults with age-related macular degeneration resulting from exposure to bright light [12]. To elaborate further, association between text size and reading speed has been explained by previous studies, where reading speed improves as text size increases [26, 27]. However, text size is not the only determinant for reading speed, other aspects such as visual angles, text spacing, and font type are important aspects that could impact one’s reading speed [26–30]. According to a study done by Pelli et al. (2007) [26], reading speed significantly increases as text size increases, but the curve of changes in reading speed starts to go flat beyond the visual threshold and toward descends with increasing text size, resulting from large visual angles that require more involvement of head and eye movement to fixate on reading target [26]. Crowding effect is a phenomenon of difficulty in recognition when a letter is surrounded by other letters. Chiu and Drieghe (2023) reported that there will be lesser crowding effect and less challenging to recognize a single letter compared to a sentence while reading at static [30]. Modified Universiti Kebangsaan Malaysia Malay Language Related Word Reading Text Test chart used in this study have enlarged the text with the same font type, Atlanta Light [19]. However, the legibility of Atlanta Light has not been investigated. Thus, we assumed that these are the factors of higher requirement in illumination level among schoolchildren with low vision when reading the reading speed test chart.

Other than that, we assumed that higher reading speed under 2000 lux is a consequence of psychological changes, as bright light could increase one’s alertness, increase excitement and vitality, improve cognitive task performance [31, 32]. Other than that, visual comfort could be one of the factors leads to high reading speed at 2000 lux. This is because visual comfort reduced at 2000 lux, and a substantial number of subjects verbally expressed that they felt glare. Thus, the subjects might tend to read in a faster pace to complete the test rapidly.

During statistical analysis, a high variation in reading speed (wpm) were noticed among the schoolchildren with low vision due to their variation in severity of vision loss. In which, schoolchildren with mild low vision read faster compared to schoolchildren with moderate low vision and severe low vision. However, the statistical findings of the relative changes in reading speed (log decimal) at different illumination levels from the baseline (125 lux) found that, all schoolchildren with low vision showed comparable improvement in reading speed with increased illumination, regardless of their degree in vision loss. Therefore, increment of illumination level in the learning space could be beneficial universally to enhance reading speed among schoolchildren with low vision.

Our previous study on illumination level of classrooms in the same school showed that the illumination level during learning ranged from 79 lux to 687 lux [33]. The average illumination level in the classroom increased gradually from 144.35 ±57.28 lux at 8 am to 249.97 ±83.85 lux (9 am), 333.75 ±106.91 lux (10 am), 395.49 ±136.55 lux (11 am), 393.91 ±142.77 lux (12 pm) and 405.09 ±139.68 lux at 1 pm [32]. These findings showed that the average illumination level in the morning (8 am to 9 am) did not meet the recommended classroom illumination by the Malaysia Standard [6]. The finding of this study showed that the classroom illuminance was not optimal for the schoolchildren’s visual functions and visual comfort and therefore did not meet their visual needs.

### Limitations

There are four special schools at secondary level for children with vision impairment in Malaysia, and the current study involved only one of the schools and therefore may not represent all. But the school in this study is the only one that offers a national standard curriculum (others offer vocational training), so the students are highly engage in reading as part of their learning activities. Furthermore, the student population is diverse and came from different states in Malaysia. Our study found that there was a significant increase in visual function and visual comfort with increased illumination level. In general, a minimum illumination of 600 lux is recommended to be adopted in the school. However, caution should be taken in implementing the recommended general lighting level because of the variety causes of vision loss. For example, increased in illumination maybe counterproductive for children with albinism who may experience significant glare. A tailored prescription of illumination can be beneficial in this case and should be made by optometrist or low vision specialist to enhance their daily visual task. Besides, unlike the examination room, the illumination level in real classroom were not constant. The real indoor environment is highly depending on the quantity, type and position of the windows and furniture or obscure, wall colour, time of the day, and amount of natural daylight from different weather condition. The physical classroom environment of the special secondary school for visually impaired were observed in this study. As the data collection was conducted during the COVID-19 pandemic, curtains were removed from all classrooms to reduce the risk of infection. Classrooms observed (n = 15) were similar in size and quantity of windows and door. Louver windows was used in all classrooms, with openwork gate door and wood door. Its noteworthy that the wall painting of each classroom were different, which include yellow, light blue, apple green, beige, grey, light purple, white, pink, and floral prints. Besides, two among the classrooms had ceramic tiles flooring of different colours, where others were cement. Unlike to most of the classrooms, the wall of examination room was painted in white, while beige ceramic tiles were used. This could be controversy if one illumination level could be applied to all classrooms, as these physical components determine the reflectance of illuminance and influence light distribution of an indoor space [6, 34–36]. Ambient lighting is a crucial factor that affect visual and cognitive perception of a person [37, 38]. However, this present study focused on the effect of illumination level on the surface of reading plane, while the effect of ambient lighting from the surrounding has not been investigated. Thus, the findings in this study may not accurately reflect the effect of illumination level on real-world conditions and how lighting interacts with reading activities.

### Future research

Future research should include a larger number of subjects, and sufficient number of subjects with the similar cause of low vision. Additional studies should replicate the illumination level in the actual classrooms, to determine and ratify the optimal illumination level after longer exposure following the school time, as schoolchildren will perform the reading activities mostly in the classroom during school time. The reading behaviour and visual needs of each schoolchild with low vision should be studied, followed by the effect of exposure time under high illuminance on visual function and visual comfort.

## Conclusion

This study showed that all visual functions which were distance and near visual acuity, distance and near contrast sensitivity, reading speed and visual comfort significantly improved across the five illumination levels among schoolchildren with low vision. Illumination level of average 600 lux, within range of 276.67 lux to 701.59 lux was recommended for reading plane in the classrooms for schoolchildren with low vision. Eye care practitioners, optometrists, architects and school authorities should take part and work together to perform necessary examinations and illumination level assessment of the schoolchildren with low vision, to maximise the visual function and visual comfort of schoolchildren with low vision by modification of lighting and physical environment of the classrooms.
